# Non-exercise Activity Thermogenesis Correlated With Clinical Parameters in Patients With or At-Risk for Chronic Obstructive Pulmonary Disease (COPD): A Pilot Study

**DOI:** 10.7759/cureus.53019

**Published:** 2024-01-26

**Authors:** Sanehiro Yogi, Toru Shirahata, Hideaki Sato, Yuki Nishida, Kaiji Inoue, Mamoru Niitsu, Tomoe Akagami, Kenji Masaki, Makoto Nagata, Shigeho Tanaka, Fuminori Katsukawa, Hidetoshi Nakamura

**Affiliations:** 1 Respiratory Medicine, Saitama Medical University Hospital, Saitama, JPN; 2 Biostatistics, M&D Data Science Center, Tokyo Medical and Dental University, Tokyo, JPN; 3 Sports Medicine Research Center, Keio University, Yokohama, JPN; 4 Radiology, Saitama Medical University Hospital, Saitama, JPN; 5 Faculty of Nutrition, Kagawa Nutrition University, Saitama, JPN

**Keywords:** body composition, physical activity, imaging analysis, non-exercise activity thermogenesis, chronic obstructive pulmonary disease

## Abstract

Background: Attention to physical activity has grown in patients with chronic obstructive pulmonary disease (COPD), as it serves as a robust indicator for mortality associated with COPD. Non-exercise activity thermogenesis (NEAT) is the energy expenditure due to physical activities besides active sports-like exercises and resistance training in daily life, and decreased NEAT may be related to physical inactivity in patients with COPD. We examined whether NEAT assessed using a questionnaire reflects clinical parameters in patients with or at risk for COPD.

Methods: The study participants consisted of 36 male patients (COPD=28; stage1=6, stage2=14, stage3/4=8, and at-risk for COPD=8) older than 50 years of age. The participants underwent anthropometric measurements, lung function testing, a six-minute walk test, muscle strength testing, and questionnaires, e.g., the COPD assessment test (CAT), modified Medical Research Council (mMRC) dyspnea scale, and Hospital Anxiety and Depression Scale. Image analysis with chest computed tomography (CT) included the number of trunk muscles, bronchial wall thickening, and emphysema (percentage of the lung ﬁeld occupied by low attenuation area <-950 HU). We evaluated the relationship between these clinical parameters and NEAT questionnaire scores using Pearson correlation analysis and the Tukey-Kramer test.

Results: The NEAT score was correlated with the severity of airflow limitation and airway wall thickness measured by chest CT, symptoms evaluated by the mMRC dyspnea scale and CAT, and inspiratory muscle strength and pectoralis muscle area assessed by CT.

Conclusion: Our study revealed the significance of NEAT as a valuable indicator in assessing the health status of patients with or at risk for COPD. The NEAT score was correlated with various clinical traits, suggesting that incorporating NEAT assessments using a questionnaire can contribute to a comprehensive understanding of the clinical condition in these patients. Further large-scale studies are warranted to validate and generalize these findings across diverse COPD populations.

## Introduction

Chronic obstructive pulmonary disease (COPD) is a progressive lung disorder characterized by irreversible airflow obstruction and alveolar destruction, mostly attributable to smoking. The degree of symptoms and functional limitations caused by COPD can differ from person to person, often resulting in delayed diagnosis. COPD also progresses with weight loss and skeletal muscle deficits [1], which limits pulmonary ventilation and increases ventilatory burden during exercise. Patients with COPD tend to avoid exercise-associated dyspnea and fatigue, leading to an inactive lifestyle. This process is called a “dyspnea-inactivity vicious cycle,” and this may happen at any stage of COPD to a certain degree [2-4].

Non-exercise activity thermogenesis (NEAT) is the energy expenditure of all physical activities other than volitional sporting-like exercise [5]. It includes various activities in daily life, such as walking to work, attending school, singing, dancing, washing clothes, and cleaning floors. Since patients with COPD are apt to have less exercise activity thermogenesis, their activity energy expenditure is considered to be mainly composed of NEAT.

We have recently reported that the total NEAT score evaluated by a questionnaire was correlated with the physical activity level (PAL) assessed by the doubly labeled water method and indirect calorimetry in patients with or at risk for COPD [6]. However, only a few previous studies have investigated the relationship between NEAT and clinical traits of COPD, including lung functions, body composition, muscle strength, and features of computed tomography (CT) images.

Therefore, we examined the NEAT score in patients with COPD using the questionnaire designed to evaluate physical activity related to NEAT and investigated how NEAT correlates with these clinical traits. We thought it might be possible to better understand the disease and develop a new treatment strategy by examining which clinical parameters are associated with the NEAT score in COPD patients.

## Materials and methods

Study design and population

In total, 37 consecutive patients with (n=28) or at risk for COPD (n=9, with chronic respiratory symptoms and smoking history≥10 pack-years, and FEV_1_/FVC≥0.7) who visited the outpatient clinic of Saitama Medical University Hospital between June 2017 and February 2018 participated in this study. COPD was defined by a post-bronchodilator FEV_1_/FVC < 70% according to the Global Initiative for Chronic Obstructive Lung Disease (GOLD) guidelines [7]. Patients at risk for COPD had similar COPD assessment test (CAT) scores to those of the patients with COPD (9.6 ± 4.0 versus 10.2 ± 6.7) and most of them (six patients) improved their symptoms by administration of inhaled bronchodilators. Exclusion criteria were infectious diseases, diabetes mellitus with medication, dysphagia, or other serious diseases that would interfere with movement; treatment with drugs affecting energy expenditure (thyroid hormone, beta-blocker, glucagon-like peptide-1 receptor agonist) or water balance (sodium-glucose cotransporter-2 blocker); and weight loss of ≥5% of their body weight (BW) during the previous three months. After one at-risk COPD patient had to discontinue the study because of acute bronchitis, the data from the remaining 36 participants were used for analysis.

This study was approved by the Institutional Review Board of Keio University on Dec 14, 2015 (Protocol No. 2015-03), Saitama Medical University Hospital on May 2, 2016 (No. 16-003-1), and the National Institutes of Biomedical Innovation, Health and Nutrition on Jan 17, 2017 (Protocol No. 29). Written informed consent was obtained from each participant.

Data collection

 All patients underwent anthropometric measurements, pulmonary function tests, a 6-minute walk test (6MWT), calf circumference (CC) measurement, hand grip strength (HGS), CAT, modified Medical Research Council (mMRC) dyspnea scale, and chest CT. The Hospital Anxiety and Depression Scale (HADS) was used to measure the emotional state of patients and their degree of anxiety and depression [8]. BW was measured to the nearest 0.1 kg using a digital scale with patients wearing light clothing. The height of each patient was measured to the nearest 0.1 cm using a horizontal headboard with an attached wall-mounted metric scale. Body mass index was calculated using these measurements (BW in kg/height in m^2^). Fat free mass and skeletal muscle mass were measured by bioelectrical impedance analysis using an SFB7 (ImpediMed, Queensland, Australia).

Assessment of NEAT using a questionnaire

 We asked participants about their daily activities to evaluate NEAT using a questionnaire (NEAT score), which was modified from a compendium of physical activities [9]. It consists of 11 questions about locomotive activities and 25 questions about non-locomotive activities [10] and is available in the online supplementary material. Each questionnaire item was evaluated with a score of 1-3 points according to the activity levels, and these points were summed to determine the total NEAT score.

Pulmonary function testing

After bronchodilator inhalation, spirometry was performed by trained and certified spirometry technicians using a FUDAC-7 instrument (Fukuda Denshi Co., Ltd., Tokyo, Japan). Lung volume subdivisions and diffusing capacity of carbon monoxide (DLco) were measured in all participants. As indices of respiratory muscle strength, maximal expiratory pressure (PEmax) and maximal inspiratory pressure (PImax) were measured with an AutospiroAS-507 spirometer (Minato Medical Science, Co., Ltd., Osaka, Japan). The predicted pulmonary function values were calculated according to the Japanese Respiratory Society equation 2014 [11].

Computed tomography scan acquisition and analysis

**Figure 1:**
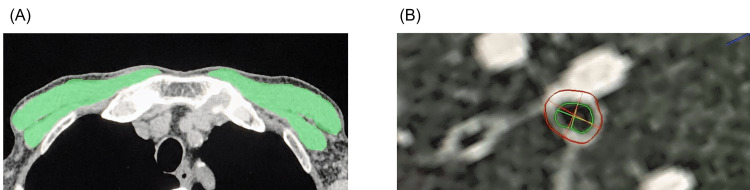
Representative cross-sectional computed tomography images. (A) The pectoralis muscle area is shown in green. (B) Lumen area (green) and wall area (between green and red) of the segmental right apical (third-generation of the segmental right apical).

Details regarding trunk muscle measurements have been provided in our previous report [12]. In brief, we performed an analysis of three trunk muscles on a single-slice axial chest CT image. The pectoralis muscle area (PMA) at the top of the aortic arch, rectus abdominis muscle area at the first lumbar vertebra, and erector spinae muscle area (ESMA) at the level of the Th12 lumbar vertebra were measured. Intra-observer agreement and inter-observer reliability were previously confirmed [11]. The percentage of low attenuation area was determined using a cutoff value of <-950 Hounsfield units. The lumen area (LA) and wall area (WA) of the segmental right apical (RB1) were automatically measured. Then, the PMA and LA were normalized to the body surface area (BSA). The wall area percentage (WA%) was calculated using the following formula: 100×WA/(sum of the LA and WA). We used SYNAPSE VINCENT volume analyzer software (FUJIFILM Medical Co., Ltd., Tokyo, Japan) for all CT image analyses. Representative cross-sectional CT images are shown in Figure 1.

Statistical analysis

The data pertaining to the continuous variables are expressed as the mean ± standard deviation (SD) unless otherwise specified. Pearson correlation analysis was used to evaluate the associations between the NEAT score and clinical parameters. To determine the extent to which the locomotive NEAT score or the non-locomotive NEAT score affects each other, a partial correlation analysis with adjustment for the non-locomotive NEAT score was performed between the locomotive NEAT score and clinical parameters, and vice versa. Multiple comparisons were performed using the Tukey-Kramer test. The Jonckheere-Terpstra trend test was performed for a trend analysis of the continuous variables. All statistical analyses were performed using the EZR 4.1.2 (R, open-source, Saitama Medical Centre, Jichi Medical University, Saitama, Japan). Statistical significance was set at p<0.05.

## Results

Patient characteristics

Table 1 presents the baseline characteristics of the study participants. All patients were male and the mean %FEV_1_ was 69.4%. The total NEAT score was 59.9 ± 8.6 (range: 40-76), the locomotive NEAT score was 18.1 ±2.6 (range: 13-25), and the non-locomotive NEAT score was 41.8 ± 7.1 (range: 26-54), respectively.

**Table 1:** Clinical biochemical and physiological data of all participants. M: Male; F: female; BMI: body mass index; GOLD: Global Initiative for Chronic Obstructive Lung Disease; VC: vital capacity; FEV_1: _forced expiratory volume in 1 second; DL_CO_/VA: diffusing capacity of carbon monoxide/alveolar volume; PImax: maximal inspiratory pressure; mMRC: modiﬁed Medical Research Council; CAT: chronic obstructive pulmonary disease assessment test; HAD-A: Hospital Anxiety and Depression Scale-Anxiety; HAD-D: Hospital Anxiety and Depression Scale-Depression; FFMI: mass index; SMI: skeletal muscle mass index; HGS: hand grip strength; CC: calf circumference; PMA: pectoralis muscle area; RAMA: rectus abdominis muscle area; ESMA: erector spinae muscle area; LA3: lumen area of the third generation bronchi; LA5: lumen area of the fifth generation bronchus; WA%3^rd^: wall area percent of the third generation bronchus; WA%5^th^:^ ^wall area percent of the fifth generation bronchus; LAA%: percentage of the lung ﬁeld occupied by the low attenuation area; HU: Hounsfield unit; NEAT: non-exercise activity thermogenesis.

Variable	Data (range)
Total number of patients	36
Sex, M/F	36/0
Age, y	70.3±5.8 (52–78)
Height, cm	165.1±5.9 (153.2–176.8)
Weight, kg	60.2±10.1 (39.8–81.0)
BMI, kg/m^2^	21.9±3.2 (15.2–30.1)
Smoking, pack-years, n	56.5±23.3 (25–120)
GOLD stage, at-risk/1/2/3/4	8/6/14/6/2
%VC, % predicted	95.3±16.2 (53.1–127.9)
FEV_1_, % predicted	69.4±24.4 (27.1–110.3)
%DLco/VA, % predicted	74.0±27.4 (28.9–127.0)
%PImax, % predicted	87.6±29.3 (33–169)
mMRC, 0/1/2/3/4	14/13/6/3/0
CAT score, n	10.1±6.1 (1–29)
HAD-A, n	3.1±2.4 (0–7)
HAD-D, n	3.9±3.0 (0–13)
6MWD, min	434.9±94.6 (275–750)
ΔSpO_2_	-7.3±4.6 (-19--1)
Borg dyspnea score, n	2.2±2.2 (0–7)
Borg fatigue score, n	0.7±1.3 (0–5)
FFMI, kg/m^2^	16.7±2.3 (11.8–20.9)
SMI, kg/m^2^	9.1±1.0 (7.1–10.7)
HGS, kg	33.9±7.0 (22.1–46.9)
CC, cm	33.3±3.1 (26.9–38.8)
PMA/BSA, cm^2^	18.6±2.9 (11.7–27.8)
RAMA/BSA, cm^2^	5.2±0.8 (3.2–7.1)
ESM/BSA, cm^2^	15.8±2.4 (8.5–20.5)
LA3/BSA, mm^2^	10.2±5.1 (1.7–21.9)
WA%3^rd^, %	59.2±8.7 (45.0–82.0)
LA5/BSA, mm^2^	3.1±1.6 (0.74–7.6)
WA%5^th^, %	66.1±7.7 (37.0–79.0)
LAA%	13.9±13.6 (0.1–52.0)
Total NEAT score	59.9±8.6 (40–76)
Locomotive NEAT score	18.1±2.6 (13–25)
Non-locomotive NEAT score	41.8±7.1 (26–54)

Distributions of the NEAT scores

The distributions of the total, locomotive, and non-locomotive NEAT scores by the GOLD stage are shown in Table 2. Although the values of patients with COPD tended to be lower as the disease severity increased, there were no significant differences among the GOLD stages. When the data of the patients classified as at-risk for COPD were excluded, trend analyses revealed that patients with higher GOLD stage displayed lower non-locomotive score (p=0.038) although there were no significant differences in the total NEAT score (p=0.069) and locomotive NEAT score (p=0.217).

**Table 2:** Distributions of the NEAT scores by the GOLD stage. NEAT: Non-exercise activity thermogenesis; GOLD: Global Initiative for Chronic Obstructive Lung Disease; COPD: chronic obstructive pulmonary disease

	At-risk for COPD	GOLD stage 1	GOLD stage 2	GOLD stages 3, 4
NEAT score, n	8	6	14	8
Total	61.5±6.4	64.8±7.7	59.9±8.6	54.5±9.7
Locomotive	18.4±3.1	18.7±2.6	18.3±2.1	17.0±2.9
Non-locomotive	43.1±5.9	46.2±5.6	41.6±7.4	37.5±7.3

Relationships between the NEAT score and clinical parameters

Table 3 presents the bivariate analysis of the total NEAT score and various clinical parameters. The total NEAT score was correlated with %FEV_1 _(r=0.419, p=0.011), percentage of velocity of expiratory airflow at 50% (%$\dot{v}$​​​​​​50) (r=0.364, p=0.029), %DLco/VA (r=0.339, p=0.043), %PImax (r=0.372, p=0.026), CAT (r=-0.342, p=0.041), mMRC dyspnea scale score (r=-0.581, p=0.0002), Borg scale (fatigue) (r=-0.354, p=0.034), PMA/BSA (r=0.364, p=0.029), and wall area percent of the fifth-generation bronchus (WA%5^th^) (r=-0.432, p=0.009).

**Table 3:** Bivariate correlation analysis of the total NEAT score and clinical parameters. BMI: Body mass index; GOLD: Global Initiative for Chronic Obstructive Lung Disease; VC: vital capacity; FEV_1_:_ _forced expiratory volume in 1 second; %$\dot{v}$_50_:percentage of velocity of expiratory airflow at 50%; SpO_2_: peripheral capillary oxygen saturation; %RV: percentage of residual volume; DL_CO_/VA: diffusing capacity of carbon monoxide/alveolar volume; mMRC: modiﬁed Medical Research Council; CAT: chronic obstructive pulmonary disease assessment test; FFMI: fat free mass index; SMI: skeletal muscle mass; HGS: hand grip strength; CC: calf circumference; PMA: pectoralis muscle area; RAMA: rectus abdominis muscle area; ESMA: erector spinae muscle area; LA3: lumen area of the third-generation bronchus; LA5: lumen area of the fifth-generation bronchus; WA%3^rd^:^ ^wall area percent of the third-generation bronchus; WA%5^th^:^ ^wall area percent of the fifth-generation bronchus; LAA%: percentage of the lung ﬁeld occupied by the low attenuation area; HU: Hounsfield unit.

Variables	r	p	Variables	r	p
Age	-0.147	0.392	Questionnaire		
Height	0.027	0.874	CAT, n	-0.342	0.041
Weight	0.168	0.326	mMRC, n	-0.581	0.0002
Pulmonary function			HAD-A, n	0.020	0.907
%VC	0.321	0.057	HAD-D, n	-0.181	0.292
%FEV1	0.419	0.011	Body composition		
*%*$\dot{v}$*_50_*	0.364	0.029	BMI, kg/m^2^	0.190	0.266
%RV	-0.137	0.425	FFMI	0.235	0.175
%DLco/VA	0.339	0.043	SMI	0.185	0.288
Muscle strength			CC, cm	0.256	0.131
HGS, kg	0.301	0.074	CT image analysis		
%PEmax	0.043	0.802	PMA/BSA, cm^2^	0.364	0.029
%PImax	0.372	0.026	RAMA/BSA, cm^2^	0.137	0.425
6MWT			ESMA/BSA, cm^2^	0.226	0.185
6MWD, m	0.301	0.074	LA3/BSA, mm^2^	0.184	0.283
ΔSpO_2_	0.047	0.786	WA%3rd	-0.248	0.145
Borg dyspnea score, n	-0.269	0.112	LA5/BSA, mm^2^	0.212	0.216
Borg fatigue score, n	-0.354	0.034	WA%5th	-0.432	0.009
			LAA%	-0.210	0.218

**Figure 2:**
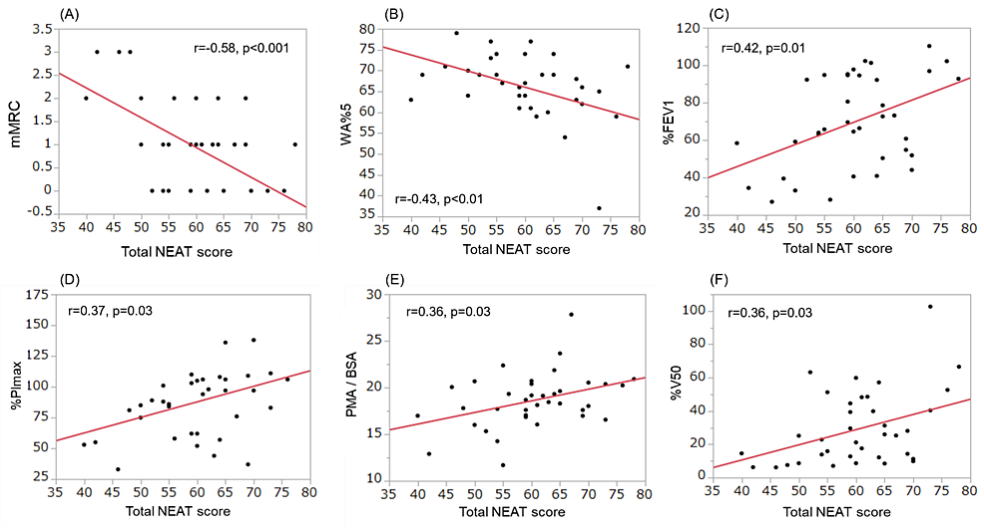
Relationship between the total NEAT score and lung function test results, computed tomography-derived cross-sectional indices (adjusted for BSA), and inspiratory muscle strength. (A) mMRC dyspnea scale, (B) WA%5^th^, (C) %FEV_1_, (D) %PImax, (E) PMA/BSA, and (F) %$\dot{v}$​​​​​​_50_ NEAT: Non-exercise activity thermogenesis; BSA: body surface area; PMA: pectoralis muscle area; FEV_1_: forced expiratory volume in 1 second

Figure 2 shows the relationships between the total NEAT scores and representative clinical parameters. When we examined the relationship between locomotive /non-locomotive NEAT scores and clinical parameters, similar trends were observed in the correlations between the non-locomotive NEAT scores and the clinical parameters, except for %DLco/VA (r=0.286, p=0.091) and %PImax (r=0.303, p=0.072). In contrast, the clinical parameters correlated with locomotive NEAT scores were limited to %DLco/VA (r=0.349, p=0.037), %PImax (r=0.411, p=0.013), 6MWD (r=0.353, p=0.035), and mMRC dyspnea scale score (r=-0.560, p=0.0004). Partial correlation analysis revealed a significant correlation between mMRC and locomotive NEAT scores (r=-0.560, p=0.012). 

## Discussion

To the best of our knowledge, this is the first report to describe the relationship between the NEAT score and clinical traits in patients with COPD. Our findings showed that the NEAT score that reflects NEAT was correlated with the severity of airflow limitation, inspiratory muscle strength, COPD-specific symptoms, and several parameters on chest CT images.

A previous study suggested that patients with COPD are physically inactive and have increased sedentary time from early stages, and the tendency became clearer as the disease worsened [4]. Although the NEAT score we obtained in this study did not show evident differences among the GOLD stages, the score was significantly correlated with %FEV_1_ in the total population. It should be noted that seven parameters, i.e., %FEV_1_, %$\dot{v}$_50_, CAT, mMRC dyspnea scale score, Borg scale score (fatigue), PMA, and WA%5^th^, were correlated with the total and non-locomotive NEAT scores, while %DLco/VA, %PImax, and mMRC were correlated with the total and locomotive NEAT scores. The 6MWD was solely correlated with the locomotive NEAT scores and not with the total and non-locomotive scores. These observations imply that airflow limitation may lead to a decrease in non-locomotive NEAT rather than locomotive NEAT, while exercise capacity may parallel locomotive NEAT.

 The NEAT score was also associated with not only %FEV_1_ but also %$\dot{v}$_50_ on lung function testing and WA%5^th^ on CT images. Since those variables are indicators of peripheral airway obstruction, this questionnaire may be useful and helpful for detecting early peripheral airway obstruction. In addition, our finding that there was no relationship between the NEAT score and the fat free mass index or skeletal muscle mass index may indicate that decreased scores on this questionnaire reflect less physical activity caused by respiratory dysfunction rather than overall muscle weakness due to aging.

In the present study, the NEAT score was also correlated with inspiratory muscle strength and PMA, which is the inspiratory accessory muscle. Respiratory muscles are essential for alveolar ventilation and work against increased mechanical loads due to impaired diaphragm function and airflow limitation. In fact, several studies have shown a correlation between respiratory muscle strength and lung function, including FEV_1_ [12-15]. Vyas et al. inferred that impaired muscle function results in a lower FEV_1_, although the mechanism remains unknown [16]. Amelioration of respiratory muscle strength has been reported to be correlated with improved physical activity, dyspnea, exercise tolerance, and disease-specific quality of life (QOL) [17], and PMA was also correlated with disease severity, QOL, exercise tolerance, and physical activity [12,18]. Therefore, if activities like inspiratory muscle training improve respiratory muscle strength and PMA, the NEAT score would increase, resulting in better exercise tolerance and improved QOL associated with the disease.

Finally, the NEAT score was correlated with COPD-specific symptoms evaluated by the mMRC dyspnea scale and CAT. The mMRC dyspnea scale score had a similar correlation with the locomotive NEAT score to that with the non-locomotive score, while CAT was only correlated with the non-locomotive NEAT score and not with the locomotive score. In other words, the mMRC dyspnea scale score reflects locomotive and non-locomotive NEAT, whereas CAT weakly and solely reflects non-locomotive NEAT.

We have recently demonstrated that the NEAT score was correlated with PAL in patients with COPD [6]. In addition, the present study indicated that the NEAT score reflected other COPD traits including airflow limitation, reduced inspiratory muscle strength and volume, and subsequent dyspnea and impaired QOL. Therefore, we believe that the NEAT score could function as a valuable and comprehensive questionnaire for assessing the health status of patients with COPD and providing personalized treatment. For example, future studies exploring the impact of rehabilitation interventions, particularly inspiratory muscle training, on NEAT scores may offer insights into improving exercise tolerance and disease-specific QOL. In addition, further investigation into the predictive value of the NEAT score for COPD prognosis may be a promising avenue for advancing patient care and management. 

This study has several limitations. First, the sample size was relatively small, and only two patients with very severe COPD were included. Second, female patients were not included because several studies demonstrated that the majority of Japanese COPD patients are male [19,20]. Therefore, the present findings may not be applicable to them. Third, the correlations between the NEAT questionnaire and COPD-related clinical parameters were not strong. This may mean the need for further refinement to tailor the questionnaire more specific to the characteristics of COPD patients.

## Conclusions

Our pilot study has provided valuable insights into the potential of the NEAT questionnaire as a comprehensive indicator in the assessment of COPD. The observed correlations with COPD-specific symptoms, inspiratory muscle strength, and peripheral airway limitations on CT imaging highlighted the multifaceted utility of the NEAT score. Incorporating the NEAT score into routine clinical practice may not only enhance our understanding of COPD but also contribute to more effective and personalized management strategies. However, the generalizability of our findings warrants validation through larger-scale studies to establish the broader applicability and reliability of the NEAT score in a diverse COPD population.

## Appendices

Non-exercise activity thermogenesis (NEAT) score

Three points are scored when subjects answer "A lot".

Two points are scored when subjects answer "Sometimes".

One point is scored when subjects answer "Hardly ever" or "No".

In question No. 4,

Three points are scored when subjects answer "Faster than most people".

Two points are scored when subjects answer "About the same as other people".

One point is scored when subjects answer "I get passed by other people".

Primarily Walking Physical Exertion

1. Do you walk on the way to work?

     A lot (over 30 minutes one way)

     Sometimes (30 minutes or less)

     Hardly ever (or do not travel to work)

2. Do you use a train or a bus (not including taxis or private cars)?

     A lot (almost every day)

     Sometimes (once or twice a week)

     Hardly ever

3. Do you walk a lot while working?

     A lot

     Sometimes

     Hardly ever (or do not work)

4. Do you walk fast or slow?

     Faster than most people

     About the same as other people

     Get passed by other people

5. Do you use stairs?

     A lot

     Sometimes

     Hardly ever (or cannot)

6. Do you go shopping for food or other daily necessities?

     A lot (once or more a day)

     Sometimes (once or more a week)

     Hardly ever

7. Do you walk to eat out (including lunch, and not including travel by car)?

     A lot (twice or more a week)

     Sometimes (about once a day)

     Hardly ever (“Hardly ever” should be less than once a week, “a lot” and “sometimes” should be 2 - 5 days a week.)

8. Do you take the garbage out?

     A lot (4 times or more a week)

     Sometimes (about once a week)

     No

9. Do you go to concerts, to the theatre or karaoke?

     A lot (once or more a week)

     Sometimes (about once a month)

     Hardly ever

10. Do you play with young children outside?

     A lot (once or more a day)

     Sometimes (once or more a week)

     No (or do not have young children)

11. Do you ever go for a walk (including walking the dog)?

     A lot (once or more a day)

     Sometimes (once or more a week)

     Hardly ever

Non-primarily Walking Physical Exertion

12. Do you ever do any light cleaning (such as picking up trash)?

     A lot (once or more a day)

     Sometimes (once or more a week)

     Hardly ever

13. Do you ever do any relatively hard cleaning (such as using a vacuum cleaner, mopping a floor, or dusting)?

     A lot (once or more a day)

     Sometimes (once or more a week)

     Hardly ever

14. Do you ever clean any large objects (such as windows, ventilation fans, or cars)?

     A lot (once or more a week)

     Sometimes (once or more a month)

     Hardly ever

15. Do you ever prepare meals (cooking or serving)?

     A lot (once or more a day)

     Sometimes (once or more a week)

     Hardly ever

16. During meals, do you often get up to fill rice bowls, or get things?

     A lot

     Sometimes

     Hardly ever

17. Do you clear the table? 

     A lot (once or more a day)

     Sometimes (once or more a week)

     Hardly ever

18. Do you wash the dishes?

     A lot (once or more a day)

     Sometimes (once or more a week)

     Hardly ever

19. Do you wipe the dishes and put them away?

     A lot (once or more a day)

     Sometimes (once or more a week)

     Hardly ever

20. Do you do the washing (carrying and putting out to dry)?

     A lot (once or more a day)

     Sometimes (once or more a week)

     Hardly ever

21. Do you bring the washing in (and fold it up)?

     A lot (once or more a day)

     Sometimes (once or more a week)

     Hardly ever

22. Do you wash the bed sheets and covers?

     A lot (every day)

     Sometimes (once or more a week)

     Hardly ever

23. Do you ever put the bedding outside in the sun?

     A lot (once or more a day)

     Sometimes (once or more a week)

     Hardly ever

24. Do you do the ironing?

     A lot (once or more a day)

     Sometimes (once or more a week)

     Hardly ever

25. Do you clean the bath and the toilet?

     A lot (once or more a day)

     Sometimes (once or more a week)

     Hardly ever

26. Do you clean the garden and around the house?

     A lot (once or more a day)

     Sometimes (once or more a week)

     Hardly ever

27. Do you ever do any weeding or gardening?

     A lot (once or more a day)

     Sometimes (once or more a week)

     Hardly ever

28. Do you water any plants?

     A lot (once or more a day)

     Sometimes (once or more a week)

     Hardly ever

29. Do you look after or feed any pets?

     A lot (once or more a day)

     Sometimes (once or more a week)

     Hardly ever

30. Do you have to look after any young children (such as cooking meals, dressing, or playing together inside)?

     A lot (once or more a day)

     Sometimes (once or more a week)

     Hardly ever

31. Do you ever pick them up (such as giving them a hug or a piggy-back ride)?

     A lot (once or more a day)

     Sometimes (once or more a week)

     Hardly ever

32. Do you have to look after anyone old or sick?

     A lot (once or more a day)

     Sometimes (once or more a week)

     Hardly ever

33. How often do you take a bath (or shower)?

     A lot (once or more a day)

     Sometimes (once or more a week)

     Hardly ever

34. Do you do any sewing or any other handcraft?

     A lot (once or more a day)

     Sometimes (once or more a week)

     Hardly ever

35. Do you play an instrument?

     A lot (once or more a day)

     Sometimes (once or more a week)

     Hardly ever

36. Do you ride a bicycle?

     A lot (once or more a day)

     Sometimes (once or more a week)

     Hardly ever
